# Right occipital transtentorial approach for a pineal malignant germ cell tumor

**DOI:** 10.3171/2021.4.FOCVID2151

**Published:** 2021-07-01

**Authors:** Brian J. A. Gill, Dominique M. Higgins, Matei A. Banu, Michael G. Argenziano, Neil A. Feldstein, Jeffrey N. Bruce

**Affiliations:** Department of Neurological Surgery, Columbia University Irving Medical Center, New York, New York

**Keywords:** pineal region, occipital transtentorial, malignant germ cell tumor, surgical video

## Abstract

Germ cell tumors account for up to 53% of the malignant lesions found in the pineal region and are typically managed with a combination of radiation therapy and chemotherapy. Malignant somatic transformation of intracranial germ cell tumors is exceedingly rare and has only been reported on two other occasions. Here the authors present the case of a pineal yolk sac tumor that failed optimum first-line treatment and underwent malignant somatic transformation to an enteric mucinous adenocarcinoma requiring surgical intervention. This video demonstrates the technical nuances of the occipital transtentorial approach and the safe microsurgical dissection of lesions within the pineal region.

The video can be found here: https://stream.cadmore.media/r10.3171/2021.4.FOCVID2151.

## Transcript

This video demonstrates the surgical resection of a malignant germ cell tumor of the pineal region via an occipital transtentorial approach.

### 0:32 Case Presentation

The patient was a 15-year-old male with no significant past medical history who initially presented with headaches and ocular findings consistent with a Parinaud’s syndrome. An MRI performed at that time showcased a large pineal region tumor. His serum alpha-fetoprotein levels were elevated; however, his serum beta–human chorionic gonadotrophin levels were within normal limits. He underwent an endoscopic biopsy and an endoscopic third ventriculostomy at an outside institution. The pathology results were consistent with a diagnosis of yolk sac tumor with enteric differentiation. After placement of an Ommaya reservoir, he started on an intrathecal chemotherapy regimen which included carboplatin, etoposide, and ifosfamide. After two rounds of induction chemotherapy, his clinical symptoms had improved. A repeat MRI scan demonstrated that his tumor had decreased in size and his AFP levels dropped markedly but were still above the normal range. However, following his sixth round of chemotherapy he started to develop worsening headaches and recurrence of his Parinaud’s. Repeat imaging demonstrated a significant increase in the size of the tumor, and his germ cell markers were notable for an interval increase in his serum AFP level.

### 1:42 Rationale for Surgical Intervention

Platinum-based chemotherapy is the initial treatment of choice for nongerminomatous germ cell tumors such as yolk sac tumors.^1^ In comparison to germinomas, nongerminomatous germ cell tumors have a higher relapse risk and a worse prognosis. As a result, multimodality therapy is often required.^2^ Candidates for delayed resection or second-look surgery include patients who fail to respond to chemotherapy, the presence of residual tumor with negative serum or CSF markers after chemotherapy and/or radiation, or suspected growing teratoma syndrome.^3^ The latter diagnosis refers to the clinical situation in which the patient has normal or declining germ cell markers, but a solitary enlarging tumor following induction chemotherapy. This occurs in 10% of nongerminomatous germ cell tumors.^4^

### 2:28 Surgical Approaches to the Pineal Region

Surgical corridors which permit access to the pineal region include the posterior interhemispheric transcallosal approach, the occipital transtentorial approach, the midline supracerebellar infratentorial approach, and the paramedian or lateral supracerebellar infratentorial approach.^5–7^ For this case, we selected the occipital transtentorial approach. This corridor permits a wider exposure of the midline and expanded working angles in addition to facilitating greater access to the inferior aspect of the lesion which extended into the cerebellomesencephalic fissure.^6^

### 2:59 Patient Positioning and Craniotomy

The patient was placed in three-point head fixation using the Mayfield skull clamp and positioned in the right lateral decubitus position to exploit gravity retraction on the ipsilateral occipital lobe. A lumbar drain may be placed prior to starting the procedure in order to facilitate dissection of the interhemispheric fissure; however, this was not required in this case as we were able to remove CSF from his Ommaya reservoir during the procedure. The head was turned inferiorly, the vertex was elevated, and the neck was flexed in order to make the falx as perpendicular to the floor as possible. The patient was registered to the neuronavigation software using the preoperative MRI. A midline sagittal incision was made over the parietal region, and a generous right parietal craniotomy was fashioned extending across the midline to the contralateral side. The dura was opened in a U-shaped fashion and elevated toward the sagittal sinus. Following removal of CSF from the Ommaya reservoir, the interhemispheric fissure is carefully dissected. Every attempt is made to preserve bridging veins, but one can be removed with safety if necessary.

### 4:05 Tentorial Opening and Tumor Dissection

The operating microscope was brought in at this time. A small incision is made in the tentorium, lateral to the straight sinus. The edges of the tentorium are then sectioned and carefully coagulated. A cottonoid is used to protect the underlying cerebellum, and monopolar cautery is then used to advance the tentorial opening through to the incisura. The arachnoid overlying the quadrigeminal cistern was then widely opened, affording us an excellent view of the dorsal surface of the tumor. Blunt dissection of the tumor capsule is then performed in order to define tissue planes around the lesion.

### 4:47 Tumor Resection

The tumor is then debulked and removed in a piecemeal fashion from the quadrigeminal plate down to the fourth ventricle. The final portion of the tumor was firmly attached along the velum interpositum. Bridging vessels in this region were cauterized and divided, and the adherent tumor was then removed. Cotton balls are used to verify the extent of tumor resection. In this manner, a radical subtotal resection was accomplished with removal of all visible tumor and an excellent view into the third ventricle at the end of the resection.

### 5:22 Postoperative Course

The patient did well neurologically. His postoperative MRI was consistent with a radical subtotal resection. A small amount of residual remained where the tumor was densely adherent to the deep venous system. The final tumor pathology was consistent with yolk sac tumor with concomitant regional malignant somatic transformation to an enteric mucinous adenocarcinoma.

### 5:43 Conclusion

In conclusion, second-look surgery is a good alternative for patients with malignant germ cell tumors which don’t respond to initial neoadjuvant treatment. The occipital transtentorial approach provides excellent exposure for pineal region tumors, particularly multicompartment lesions, as illustrated in this case.

Microsurgical techniques and internal debulking while paying attention to preserving the tumor brain interface permits aggressive resection of these lesions.

## Author Contributions

Primary surgeon: Feldstein, Bruce. Assistant surgeon: Gill. Editing and drafting the video and abstract: Gill, Higgins, Banu, Argenziano, Bruce. Critically revising the work: Gill, Higgins, Banu, Argenziano, Bruce. Reviewed submitted version of the work: all authors. Approved the final version of the work on behalf of all authors: Gill. Supervision: Feldstein, Bruce.
